# Genome Sequence of *Stenotrophomonas maltophilia* Strain AU12-09, Isolated from an Intravascular Catheter

**DOI:** 10.1128/genomeA.00195-13

**Published:** 2013-05-02

**Authors:** Li Zhang, Mark Morrison, Páraic Ó Cuív, Paul Evans, Claire M. Rickard

**Affiliations:** Health Practice Innovation, Griffith University, Brisbane, Australiaa;; CSIRO Livestock Industries, Queensland BioScience Precinct, Brisbane, Australiab

## Abstract

*Stenotrophomonas maltophilia* is an opportunistic nosocomial pathogen that is characterized by its high-level intrinsic resistance to a variety of antibiotics and its ability to form biofilms. Here, we report the draft genome sequence of *Stenotrophomonas maltophilia* AU12-09, isolated from an intravascular catheter tip.

## GENOME ANNOUNCEMENT

*Stenotrophomonas maltophilia* was first isolated in 1943 as *Bacterium bookeri* and was then named *Pseudomonas maltophilia* (1). Later, however, investigators using rRNA cistron analysis determined that this microorganism was more appropriately named *Xanthomonas maltophilia*. More recently, DNA-rRNA hybridization studies and sequencing and mapping of PCR-amplified 16S rRNA genes have resulted in the classification and naming of *X. maltophilia* as *Stenotrophomonas maltophilia* (2). *S. maltophilia* is predominantly found in an aquatic or humid environment, and in hospitals, *S. maltophilia* is found as a contaminant of numerous medical devices, edetic acid anticoagulant in vacuum tubes for blood collection, chlorhexidine-cetrimide disinfectant, and sterile water (3).

*S. maltophilia* is the third most common nosocomial nonfermenting Gram-negative bacillus (4). A recent study shows that 4.3% of almost 75,000 Gram-negative infections were caused by *S. maltophilia* in intensive care units in the United States (5). The two most common diseases caused by *S. maltophilia* are bacteremia and pneumonia, with infection being via an intravascular catheter or ventilator, respectively (5). In addition, *S. maltophilia* is intrinsically resistant to a variety of clinically prescribed antibiotics (3). Also, *S. maltophilia* can form biofilms, which further increases its resistance to phagocytes and antibiotics (3).

*S*. *maltophilia* strain AU12-09 was isolated from an intravascular catheter tip by rolling the tip back and forth on the surface of a Columbia agar plate supplemented with 5% sheep blood, essentially as described by Maki et al. (6). DNA was prepared and the genome sequence of *S. maltophilia* AU12-09 was determined on a 454 GS FLX system using titanium chemistry (Roche) (7). The sequence data consist of 129,784,052 bp of DNA sequence at 29× coverage. A total of 125 contigs (>500 bp) were *de novo* assembled using the Roche GS *de novo* assembler (version 2.3). The contig N_50_ was 69,081 bp, and the largest contig assembled was 320,581 bp. The contigs were then ordered and oriented into four scaffolds using paired-end information. The average length of the scaffolds was 1,145,290 bp.

The draft genome of *S. maltophilia* AU12-09 consists of a circular 4,547,300-bp chromosome with a G+C content of 66.5%. The genome was automatically annotated using the RAST server (8). The genome contains 70 tRNA genes coding for all amino acids and 4,004 predicted protein-coding genes, consistent with other sequenced *Stenotrophomonas* spp. (5, 9). We identified numerous putative virulence factors, including those involved in quorum sensing, biofilm formation, and the production of bacteriocins and invasins. The *S. maltophilia* AU12-09 genome contains 24 genes coding for multidrug resistance efflux pumps, 11 genes coding for resistance to beta-lactam antibiotics, 5 genes coding for multidrug resistance tripartite systems, and 4 genes coding for resistance to fluoroquinolones.

This sequence information for the *S. maltophilia* genome will greatly improve our understanding of the drug resistance and pathogenicity of this organism.

### Nucleotide sequence accession number.

The genome sequence of *S. maltophilia* AU12-09 has been deposited in NCBI GenBank under accession no APIT00000000.
